# Effects of childhood trauma experience and COMT Val158Met polymorphism on brain connectivity in a multimodal MRI study

**DOI:** 10.1002/brb3.1858

**Published:** 2020-09-30

**Authors:** Tian Tian, Jia Li, Guiling Zhang, Jian Wang, Dong Liu, Changhua Wan, Jicheng Fang, Di Wu, Yiran Zhou, Wenzhen Zhu

**Affiliations:** ^1^ Department of Radiology Tongji Hospital Tongji Medical College Huazhong University of Science and Technology Wuhan China; ^2^ Tongji Medical College Huazhong University of Science and Technology Wuhan China

**Keywords:** catechol‐O‐methyltransferase, childhood adversity, functional connectivity, graph theory, morphometric similarity network, RRID: SCR_001847, RRID: SCR_007037, RRID: SCR_009446, RRID: SCR_009487, RRID: SCR_009550

## Abstract

Childhood adversity may act as a stressor to produce a cascade of neurobiological effects that irreversibly alter neural development, setting the stage for developing psychopathology in adulthood. The catechol‐O‐methyltransferase (COMT) Val158Met polymorphism has received much attention as a candidate gene associated with environmental adversity, modifying risk for psychopathology. In this study, we aim to see how gene × brain × environment models give a more integrative understanding of brain modifications that contribute to predicting psychopathology related to childhood adversity. A large nonclinical sample of young adults completed Childhood Trauma Questionnaire (CTQ), behavioral scores, multimodal magnetic resonance imaging (MRI) scans, and genotyping. We utilized graph‐based connectivity analysis in morphometric similarity mapping and resting‐state functional MRI to investigate brain alterations. Relationships among COMT genotypes, CTQ score, imaging phenotypes, and behavioral scores were identified by multiple regression and mediation effect analysis. Significant main effect of CTQ score was found in anatomic connectivity of orbitofrontal cortex that was an outstanding mediator supporting the relationship between CTQ score and anxiety/harm‐avoiding personality. We also noted the main effect of childhood trauma on reorganization of functional connectivity within the language network. Additionally, we found genotype × CTQ score interactions on functional connectivity of the right frontoparietal network as well as anatomic connectivity of motor and limbic regions. Our data demonstrate childhood adversity and COMT genotypes are associated with abnormal brain connectivity, structurally and functionally. Early identification of individuals at risk, assessment of brain abnormality, and cognitive interventions may help to prevent or limit negative outcomes.

## Graphical abstract

Childhood adversity leads to experience‐dependent brain modification of anatomic connectivity in the medial orbitofrontal cortex. Childhood adversity leads to experience‐dependent brain reorganization of functional connectivity within the language network. Interactive effects between childhood trauma and the COMT Val158Met polymorphism suggest that the Met heterozygote contributes to negative effects of childhood trauma on brain connectivity involving motor performance, emotion regulation, executive function, and cognitive control.

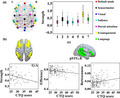

## 1 INTRODUCTION

Childhood trauma experience includes emotional abuse and neglect, physical abuse and neglect, and sexual abuse. It impacts a child's cognitive performance and emotional behavior during development (Dauvermann & Donohoe, 2019; Dvir et al., 2014). Even healthy adults who are victims in childhood remain at elevated risk for psychological problems. Researchers have proposed multiple potential mechanisms by which childhood trauma experience increases risk for psychiatric disorders. Associations among early trauma, abnormal brain development, and psychopathology are compelling. Childhood trauma experience can act as a life stressor to produce a series of hormonal changes and physiological reactions that cause overstimulation to neurons during early sensitive or critical periods, resulting in permanent modification to the neural structure and function (Teicher, 2002).

Emerging neuroimaging evidence intuitively indicates altered brain development trajectories related to childhood trauma experience, which regulate vulnerability for developing mental disorders later in adulthood (Hart & Rubia, 2012; Teicher et al., 2016), such as posttraumatic stress disorder (Klaming et al., 2019), anxiety (Ahmed‐Leitao et al., 2019), depressive disorders (Opel, et al., 2019), substance abuse (De Bellis et al., 2019), antisocial behavior (Busso et al., 2017), and personality disorders (Nicol et al., 2015). Regional alterations involving cognitive and emotional functions, such as the amygdala, hippocampus, prefrontal cortex, insular, and cingulate gyrus, are consistently supported (Cancel et al., 2019; Klaming et al., 2019; Opel, et al., 2019; Opel, et al., 2019; van Velzen et al., 2016). Moreover, a large number of literatures have reported early trauma‐related alterations in the specific pathways or network architectures that govern conscious perception, emotion regulation, threat detection, defense response, and reward anticipation, including default mode network (Bluhm et al., 2009), emotion circuitry (Cisler et al., 2018), limbic network (Cisler, 2017; Souza‐Queiroz et al., 2016), salience network (van der Werff et al., 2013), visual‐limbic fiber pathway (Choi et al., 2012), and global white matter network (Puetz et al., 2017). Overall, imaging study is a perfect way to noninvasively explore brain response to environmental effects in vivo. Knowledge of which brain development trajectories are altered by childhood trauma is the key to understanding the pathogenesis and development process, facilitating better intervention strategies for trauma‐exposed individuals.

Brain development may be modified by childhood trauma experience but fundamentally directed by genes. Genetic predisposition can influence the effects of environmental adversity on brain structure and function. On this basis, the interactions of genetic factors and childhood trauma experience may explain the occurrence and development of psychiatric symptoms. The possibility that childhood trauma experience is a stimulus to the genetic risk for psychopathology is accounted in many researches (Caspi et al., 2003; de Castro‐Catala et al., 2017; Vrijsen et al., 2014), underlying possible mechanisms on elevated risk for stress‐related response (Grabe et al., 2016), aggression (Holz et al., 2016), depression (Opel, et al., 2019), and anxiety (van Velzen et al., 2016) in adulthood. In fact, almost mental disorders have potential mechanisms and emerge through the interplay of environmental and genetic factors.

Dopamine is an important neurotransmitter that plays critical roles in response to life stress. Dopaminergic genes are widely recognized in pathophysiological mechanisms and related to psychosis proneness in individuals with traumatic experience (Green et al., 2014; Kotowicz et al., 2019). The catechol‐O‐methyltransferase (COMT), a research hotspot in dopamine system, catalyzes the degradation of synaptic dopamine in the brain. The COMT gene contains a crucial single nucleotide polymorphism (Val158Met) that results in a fourfold decrease in enzymatic activity at body temperature in Met allele carriers (Mannisto & Kaakkola, 1999). Decreased enzymatic activity leads to increased synaptic dopamine concentrations, which may affect cognitive and emotional functions via modulation of brain structure and function (Barnett et al., 2008; Gennatas et al., 2012; Mier et al., 2010). Interestingly, the COMT Val158Met polymorphism has received much attention as a candidate gene associated with environmental adversity in recent psychosis researches. A number of studies have reported that carriers of the Met allele are differentially affected by the environment, by showing a greater stress hormone response (Oswald et al., 2004; van Winkel et al., 2008). Several studies have investigated the interactions of the COMT Val158Met genotype and childhood trauma on substance abuse, suicidal behavior, schizotypal traits, anxiety, and affective disorders (Bernegger et al., 2018; Klauke et al., 2012; Savitz et al., 2010; Vinkers et al., 2013), providing evidence for gene–environment interaction mechanisms in the formation of psychotic symptoms. Further, a functional magnetic resonance imaging (MRI) study reveals that hippocampal activation mediates the relationship among childhood trauma, COMT Val158Met polymorphism, and psychiatric risk (van Rooij et al., 2016). Gene × brain ×environment models of large samples are imperative for enhancing knowledge about the etiology, prevention, and customized therapeutic directions of mental disease in trauma‐exposed individuals. But to date, relatively little research has utilized multimodal MRI to explore interactions between childhood trauma and the COMT Val158Met polymorphism on both brain anatomic and functional modifications.

Recently, many researchers have focused on exploring the nature of the brain's intrinsic activity by examining the modern network analysis based on graph theory. The large‐scale topological analyses of these coherent spontaneous brain activities highlight many important statistical characteristics underlying structural and functional connectivity in the human brain. Combined graph‐based connectivity analysis of structural and functional MRI is more appropriate to provide a complete picture of imaging phenotypes (He & Evans, 2010). Morphometric similarity mapping is a new method to examine the cortical connectivity generated from structural MRI (Seidlitz et al., 2018). Rather than estimating a single morphometric feature (like cortical thickness or volume) measured in the structural covariance network, morphometric similarity mapping extracts multiple different anatomic indices at each cortical area and estimates the Pearson product–moment correlation coefficient for each regional pair of MRI feature vectors. These pairwise measures of morphometric similarity are compiled to construct a morphometric similarity matrix, also known as morphometric similarity network (MSN). As a useful tool to analyze psychologically relevant biological mechanisms, MSN topology can predict individual difference in cognition (Seidlitz et al., 2018) and abnormal cortical patterning in psychosis (Morgan et al., 2019). Graph‐based connectivity analysis is popular in functional MRI studies and can be utilized to investigate network properties that reveal specific biological functions in interconnected subsystems, such as the sensorimotor, language, attention, visual, salience, frontoparietal, and default mode networks. These quantifiable network properties provide important implications for normal development and diseases (Gargouri et al., 2016; Tomson et al., 2015; Wang, et al., 2015; Xie & He, 2011). In this study, we recruited a large nonclinical sample of young adults that completed Childhood Trauma Questionnaire (CTQ), behavioral scores, multimodal MRI scans, and genotyping. We combine graph‐based connectivity analysis of structural and functional MRI to give a more integrative understanding of brain modifications influenced by childhood trauma experience and the COMT Val158Met polymorphism. We focus on: (a) which areas of the brain are affected by childhood trauma experience; (b) whether brain alterations mediate the environment–brain–behavior pathway; and (c) whether the COMT Val158Met polymorphism regulates the effects of childhood trauma experience on brain connectivity and behavior. Understanding the effects of childhood trauma experience and COMT Val158Met polymorphism on abnormal brain connectivity contributes to predicting the occurrence and development of psychopathology. Early identification of individuals at risk, assessment of brain abnormality, and cognitive interventions may help to prevent or limit negative outcomes.

## 2 MATERIALS AND METHODS

### 2.1 Participants

We recruited a total of 216 nonclinical samples (aged 20–30 years) from the community. All subjects completed a structured clinical interview with the Chinese version of the Mini International Neuropsychiatric Interview. Participants were carefully screened to ensure that they had no histories of psychosis, psychiatric treatment, somatic disease potentially affecting the brain, substance abuse, and MRI contraindications. Only Chinese Han populations were included to purify the sample. All subjects were strongly right‐handed according to the Chinese edition of the Edinburgh Handedness Inventory. The human experiment was approved by the Ethical Committee of Tongji Hospital of Tongji Medical College of Huazhong University of Science and Technology. All subjects gave written informed consent in accordance with the Declaration of Helsinki. All methods were carried out in accordance with approved institutional guidelines and regulations.

### 2.2 Questionnaires

In the present study, maltreatment experience during childhood was assessed by administering the Chinese version of the CTQ that assesses childhood experience of abuse or neglect in adults (Bernstein et al., 1997). The CTQ‐Short Form, a version of the CTQ that has been proven as a standardized and adequately validated implement for retrospective assessment of childhood adversity (Everaerd et al., 2016), is a 28‐item self‐report questionnaire designed to assess five types of childhood maltreatment, namely emotional abuse, emotional neglect, sexual abuse, physical abuse, and physical neglect. Participants also completed Tridimensional Personality Questionnaire (TPQ) and Spielberger's State‐Trait Anxiety Inventory (STAI) to further characterize subjects.

### 2.3 Genotyping

DNA was extracted using a DNeasy Blood & Tissue Kit. The single nucleotide polymorphism, COMT rs4680 (Val158Met), was genotyped with technical support from the Shanghai Biotechnology Corporation. Exome capture was performed using the SureSelect Human All Exon V6 (Agilent Technologies) according to the manufacturer's instructions. The quantity of libraries was assessed by Qubit^®^ 2.0 Fluorometer. The quality and size of libraries were measured by 2,100 Bioanalyzer High Sensitivity DNA Assay according to the Reagent Kit Guide. For Illumina sequencing, the qualified libraries were applied to 2 × 150 bp paired‐end sequencing on Illumina HiSeq X Ten platform (Illumina). Thirty‐nine subjects were excluded from further analysis due to the low quality of blood samples. The COMT Val158Met genotype distribution in the sample (177 subjects) was in Hardy–Weinberg equilibrium (*p* > .05). The frequencies of the COMT genotypes are presented in Table 1.

**Table 1:** Demographic and behavioral characteristics of the sample

Demographics	Total (*N* = 177)	Val/Val (*N* = 93)	Val/Met (*N* = 75)	Met/Met (*N* = 9)
	Mean (range)	Mean (range)	Mean (range)	Mean (range)
Age (years)	24.0 (20–30)	23.7 (20–30)	24.2 (20–29)	25.0 (23–27)
Gender (female/male)	130/47	69/24	56/19	5/4
CTQ sum score	30.1 (25–48)	30.2 (25–48)	29.9 (25–44)	30.0 (25–37)
STAI score	69.2 (42–119)	69.2 (42–111)	68.8 (46–119)	73.2 (61–87)
TPQ				
Novelty seeking	14.0 (4–29)	13.7 (4–23)	14.3 (5–29)	13.8 (8–19)
Harm avoiding	15.1 (2–30)	14.7 (2–28)	15.9 (4–30)	13.0 (8–19)
Reward depending	19.1 (10–27)	19.4 (10–27)	18.6 (11–27)	19.7 (16–24)

Abbreviations

CTQ, Childhood Trauma Questionnaire; STAI, State‐Trait Anxiety Inventory; TPQ, Tridimensional Personality Questionnaire.

### 2.4 MRI and data preprocessing procedure

All scans were performed on a 3.0‐Tesla MR System (Discovery MR750, General Electric, Milwaukee, WI, USA). Tight but comfortable foam padding was used to minimize head motion, and earplugs were used to reduce scanner noise. Resting‐state functional MRI (fMRI) data were obtained using single‐shot echo‐planar imaging (SS‐EPI) with the following parameters: repetition time (TR)/echo time (TE) = 2,000/30 ms; field of view (FOV) = 220 mm × 220 mm; matrix = 64 × 64; flip angle (FA) = 90°, slice thickness = 3 mm; no gap; 36 interleaved transverse slices; 185 volumes. During the fMRI scans, all subjects were instructed to keep their eyes closed, to relax and move as little as possible, to think of nothing in particular, and to not fall asleep. Sagittal 3D T1‐weighted images were acquired using a brain volume (BRAVO) sequence (TR/TE = 8.16/3.18 ms; inversion time = 450 ms; FA = 12 degree; FOV = 256 mm × 256 mm; matrix = 256 × 256; slice thickness = 1 mm; no gap; 188 sagittal slices).

We used FreeSurfer v6.0.0 software for the structural data preprocessing pipeline (RRID: SCR_001847; http://surfer.nmr.mgh.harvard.edu). Briefly, the cortical surface for each participant was reconstructed from 3D T1‐weighted images by the following steps: skull stripping, tissue classification, surface extraction, and cortical parcellation. All scans were quality controlled to reduce the impact of motion artifacts.

The resting‐state fMRI data were preprocessed using SPM8 (RRID: SCR_007037; http://www.fil.ion.ucl.ac.uk/spm). The first 10 volumes for each subject were discarded to allow the signal to reach equilibrium and the participants to adapt to the scanning noise. The remaining 175 volumes were then corrected for the acquisition time delay between slices. All subjects' fMRI data were within the defined motion thresholds (translational or rotational motion parameters lower than 2 mm or 2°). The approach used to normalize these functional images included the following steps: (a) Individual structural images were linearly coregistered to the mean functional image after motion correction; (b) the transformed structural images were segmented into gray matter, white matter, and cerebrospinal fluid; and then gray matter was nonlinearly coregistered to the Montreal Neurological Institute (MNI) space; and (c) the motion‐corrected functional volumes were spatially normalized to the MNI space using the parameters estimated during nonlinearly coregistration. The functional images were then resampled into a voxel size of 3 × 3 × 3 mm^3^. After normalization, images were smoothed using a Gaussian kernel of 8 × 8 × 8 mm^3^ full width at half‐maximum. The datasets were band‐pass‐filtered with frequency from 0.01 to 0.1 Hz, and several nuisance covariates (six motion parameters and average blood oxygen‐level‐dependent signals of the ventricular and white matter) were regressed out from the data.

### 2.5 MSN mapping and cytoarchitectonic classes

The cortical parcellation was created in FreeSurfer standard anatomic template (fsaverage) space using the Desikan–Killiany–Tourville atlas with 31 cortical regions per hemisphere (see Table S1) and subsequently transformed to each individual subject's surface. For each region, we estimated seven surface‐ and volume‐based features from the MRI data: gray matter volume, surface area, cortical thickness, Gaussian curvature, mean curvature, curved index, and folding index. Each of the MRI feature vectors in each region was normalized (z‐scored). Then, we estimated Pearson's correlation for each pair of z‐scored morphometric feature vectors. These pairwise measures of morphometric similarity were compiled to construct a morphometric similarity matrix which was known as MSN (Morgan et al., 2019). Graph theory analyses were performed by utilizing Gretna software (RRID: SCR_009487; http://www.nitrc.org/projects/gretna/) (Wang, et al., 2015) and visualized by using BrainNet Viewer software (RRID: SCR_009446; http://www.nitrc.org/projects/bnv/) (Xia et al., 2013). To characterize the topologic organization of the MSN, we calculated the following measures: global efficiency, local efficiency, clustering coefficient, the shortest path length, and small‐world parameters. For regional nodal characteristics, we considered the nodal efficiency, betweenness centrality, and degree.

To test the correspondence between morphometric similarity and cytoarchitecture, each of the 62 regions in the cortical parcellation scheme was assigned to a cytoarchitectural class using an independent modular decomposition as proposed in the previous study (Seidlitz et al., 2018). Thus, we manually assigned our MSN nodes to the seven cortical classes: primary motor cortex (class 1), association cortex (classes 2 and 3), primary/secondary sensory cortex (class 4), primary sensory cortex (class 5), limbic regions (class 6), and insular cortex (class 7).

### 2.6 Graph theory and network topological metrics in fMRI

We computed functional connectivity and topological metrics in the Conn connectivity toolbox (RRID: SCR_009550; http://www.nitrc.org/projects/conn) running on MATLAB (Whitfield‐Gabrieli & Nieto‐Castanon, 2012). Thirty regions of interest (ROIs) from Conn network cortical atlas represented 7 common networks: default mode network (4 ROIs), sensorimotor network (3 ROIs), visual network (4 ROIs), salience network (7 ROIs), dorsal attention network (4 ROIs), frontoparietal (4 ROIs), and language network (4 ROIs) (see Table S2). We calculated the functional connectivity between each pair of the 30 ROIs by computing Pearson's correlation coefficient between the two averaged time series. The resulting correlation was later transformed to approximate a Gaussian distribution using Fisher's r‐to‐z transformation. Thus, for each subject, we obtained a 30 × 30 correlation matrix, with each element representing the connectivity strength between the corresponding two ROIs. For graph analysis, the correlation matrix of ROI‐to‐ROI connectivity values was thresholded at a fixed network‐level correlation coefficient value (0.15) to define an undirected weighted graph. The topological metrics of network characteristics included the global efficiency, local efficiency, betweenness centrality, the average path length, clustering coefficient, and degree. The nodal characteristics mainly included the nodal degree, efficiency, and betweenness centrality.

### 2.7 Statistical analysis

Statistical analyses for the demographic and behavioral data were performed using Statistical Package for the Social Sciences version 18.0 (SPSS) for Windows. The whole scale's reliability was checked with Cronbach's alpha coefficient. The Kolmogorov–Smirnov test was applied to determine whether imaging and behavioral data satisfied normal distribution. A chi‐square test was conducted to test whether there were significant gender differences in the genotypic distribution. Two‐sample *t* test was utilized to test whether there were significant gender differences in CTQ score, age, TPQ score, and anxiety score. We used a multiple linear regression framework (Aliev et al., 2014) with age and sex as covariates to test the main effect or interaction between COMT genotype and CTQ score on imaging and behavioral data. Characteristics computed on each node/class/network were set as the dependent variable. Considering that many indices of anatomic and functional properties were analyzed as dependent variables simultaneously, the Bonferroni correction was used for multiple comparisons (**p < .001). If the results cannot pass the strict correction, an uncorrected threshold (*p < .05) was used to show the trend of significance. Scatter diagram was used to show the effect of CTQ score on imaging data visually. If the interaction was significant, linear correlations between CTQ score and imaging data were presented in scatter diagrams to visually display linear regression findings in different COMT genotypes, respectively. Analysis of variance was used to explore significant differences in mean nodal similarity between cytoarchitectonic classes or functional connectivity between functional networks, respectively. The Bonferroni correction was used for comparisons among groups.

### 2.8 Mediation effect analysis

A three‐variable mediation model is used to identify the mechanism that underlies the relationship between an independent variable X and a dependent variable Y via introducing a mediator variable M, which may improve our understanding of the relationship between variables even when the variables appear to be lack of a direct connection. In this study, we used the SPSS macro to perform the mediation analysis (http://www.processmacro.org/index.html). We defined the CTQ score as the independent variable X, the imaging data (showing significant main effect of CTQ score or interaction effect) as the mediator variable M, and the behavioral data (STAI/TPQ scores) as the dependent variable Y. If there was an interaction effect on the imaging data, we respectively defined variables in each COMT genotype. The first step was to confirm that the independent variable X is a predictor of the dependent variable Y, which is known as the direct effect. The second step was to confirm that the independent variable X is a predictor of the mediator M. The third step was to confirm that the mediator M is a predictor of the dependent variable Y, while controlling for the independent variable X. Path “a” represents the effect of the predictor X on the mediator M; path “b” represents the association of the mediator M with the dependent variable Y; path “*c*′” represents the direct effect of the predictor X on the dependent variable Y; and path “*c*” represents the total effect of the predictor X on the dependent variable Y. The indirect effect “*ab*” is the product of path coefficients of the last 2 steps. We used the bootstrapping method to assess the significance of the mediation effect. According to the distribution of the indirect effect after 5,000 bias‐corrected bootstrapping, we calculated 95% confidence intervals for the effect. If zero did not fall between the resulting 95% confidence interval, we concluded that there was a significant mediation effect (*p* < .05).

## 3 RESULTS

### 3.1 Demographic and behavioral characteristics

Demographic and behavioral characteristics of the sample (*N* = 177) are summarized in Table 1. Cronbach's alpha value for the whole scale was satisfactory (*α* = 0.727). Imaging characteristics and behavioral scores that set as the dependent variable satisfied normal distribution. Significant main effect of CTQ score was found on STAI score (*F* = 5.984, **p < .001, *R* ^2^ = 0.149; *B* = 0.418, *t* = 4.530, **p < .001). There was no significant main effect or genotype × CTQ score interaction on age and TPQ score (*p* > .05). There were no significant gender differences in genotypic distribution, CTQ score, age, TPQ score, and anxiety score (*p* > .05).

### 3.2 Main effect of CTQ score and interactions on MSN

**Figure 1:**
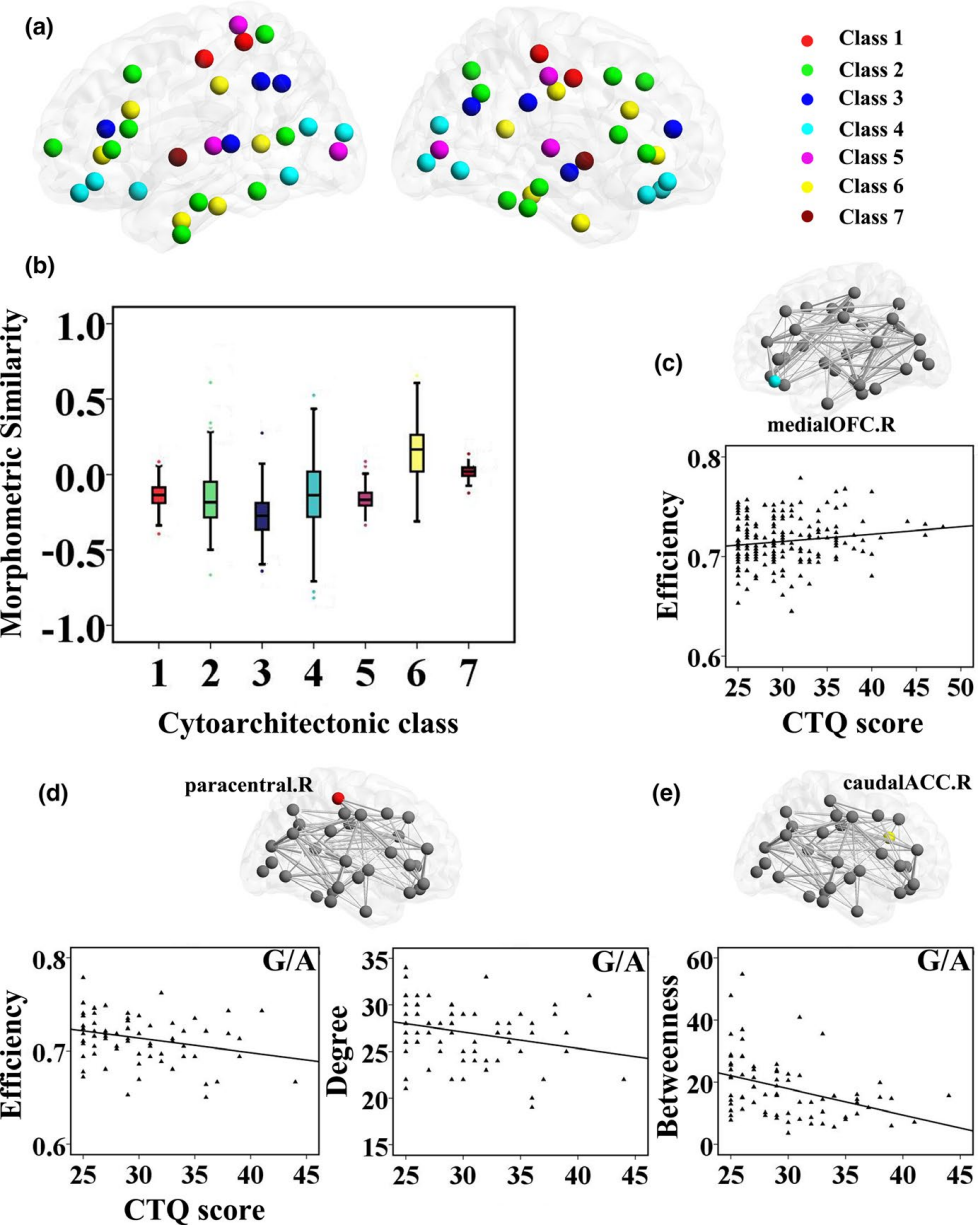
Main effect of CTQ score and interactions on MSN related to the cytoarchitectonic classification. By aligning MSN with the cytoarchitectonic classes (a), there were significant differences in mean nodal similarity between classes (b). At the nodal level of analysis (threshold with a sparsity of 10%), we found significant main effect of the CTQ score on efficiency of the right medial OFC in the class 4; the CTQ score was positively associated with efficiency of the right medial OFC (c). Additionally, we found significant genotype × CTQ score interactions on efficiency of the right paracentral lobule in the class 1, degree of the right paracentral lobule in the class 1, and betweenness of the right caudal ACC in the class 6. The CTQ score was negatively associated with nodal characteristics of the right paracentral lobule (d) and the right caudal ACC (e) in the G/A genotype. ACC: anterior cingulate cortex; CTQ: Childhood Trauma Questionnaire; MSN: morphometric similarity network; OFC: orbitofrontal cortex; R: right

We explored the distribution of nodal similarity in MSN related to the cytoarchitectonic classification (Figure 1a). Cytoarchitectonic classification is a well‐established way of evaluating histological similarity between cortical areas. By aligning MSN with the classical cytoarchitectonic atlas, we found significant differences in mean nodal similarity between classes (*F* = 143.726, **p < .001) (Table S3). Cytoarchitectonic classes 6 and 7 (corresponding to limbic regions and insular cortex) comprised cortical areas with a higher nodal similarity than the other cytoarchitectonic classes (corresponding to association, motor, and sensory cortical areas) (Figure 1b). There was no significant main effect or genotype × CTQ score interaction on mean similarity, efficiency, degree, and betweenness of each class (*p* > .05). At the nodal level of analysis (threshold with a sparsity of 10%), we found significant main effect of the CTQ score on efficiency of the right medial orbitofrontal cortex (OFC) in the class 4 (*F* = 2.316, *p = .046, *R* ^2^ = 0.063; *B* = 0.209, *t* = 2.159, *p = .032). The CTQ score was positively associated with efficiency of the right medial OFC in the scatter diagram (*r* = 0.153, *p = .042) (Figure 1c).

Furthermore, genotype × CTQ score interactions were found in efficiency of the right paracentral lobule in the class 1 (*F* = 2.691, *p = .023, *R* ^2^ = 0.073; *B* = −0.149, *t* = −3.406, *p = .001), degree of the right paracentral lobule in the class 1 (*F* = 2.309, *p = .046, *R* ^2^ = 0.063; *B* = −0.169, *t* = −3.090, *p = .002), and betweenness of the right caudal anterior cingulate cortex (ACC) in the class 6 (*F* = 4.353, *p = .001, *R* ^2^ = 0.113; *B* = −0.086, *t* = −4.127, **p < .001). In the scatter diagrams (Figure 1d), the CTQ score was negatively associated with efficiency (*r *= −0.280, *p = .015) and degree (*r* = −0.262, *p = .023) of the right paracentral lobule in the G/A genotype (Val/Met heterozygote). Similarly, in Figure 1e, the CTQ score was negatively associated with betweenness of the right caudal ACC in the G/A genotype (*r* = −0.388, *p = .001). There were no significant correlations between CTQ score and network properties of the right paracentral lobule/the right caudal ACC in the other genotypes (*p* > .05) (see Figure S1).

### 3.3 Main effect of CTQ score and interaction on fMRI networks

**Figure 2:**
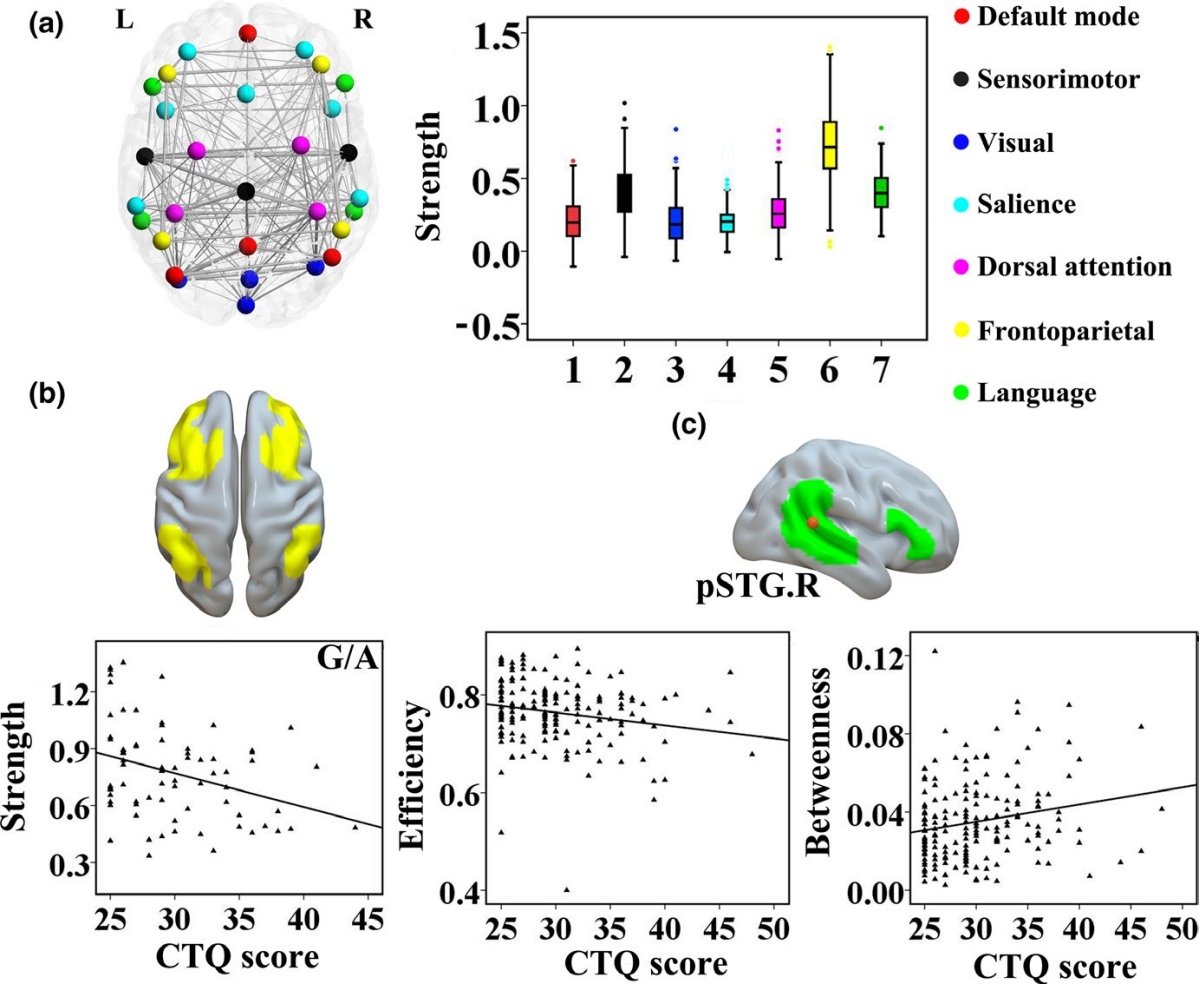
Main effect of CTQ score and interaction on functional connectivity of 7 classical networks (a). Distribution of nodes from 7 classical networks in the fMRI graph theory analysis is presented in Figure 2a; there were significant differences in mean functional connectivity strength between networks. We found significant interaction on connectivity strength of the right frontoparietal network; the CTQ score was negatively associated with connectivity strength in the G/A genotype (b). At the nodal level of analysis, there were significant main effects of CTQ score on efficiency and betweenness of the right pSTG within the language network; the CTQ score was negatively related to efficiency of the right pSTG and positively associated with betweenness of the right pSTG (c), respectively. CTQ: Childhood Trauma Questionnaire; fMRI: functional magnetic resonance imaging; L: left; pSTG: posterior superior temporal gyrus; R: right

Distribution of nodes from 7 classical networks in the fMRI graph theory analysis is presented in Figure 2a. We found significant differences in mean functional connectivity between networks (*F* = 305.206, **p < .001) (Table S4). The frontoparietal network with a higher mean strength than the others is observed in Figure 2a. There was no significant main effect or genotype × CTQ score interaction on the mean topological metrics of each network (*p* > .05). For mean connectivity strength within each network, we found interaction on connectivity strength of the right frontoparietal network (*F* = 2.380, *p = .041, *R* ^2^ = 0.065; *B* = −0.395, *t* = −2.543, *p = .012). In Figure 2b, the CTQ score was negatively associated with strength of the right frontoparietal network in the G/A genotype (*r* = −0.333, *p = .004). There were no significant correlations between CTQ score and strength of the right frontoparietal network in the other genotypes (*p* > .05) (see Figure S2). At the nodal level of analysis, there were main effects of CTQ score on efficiency (*F* = 2.725, *p = .021, *R* ^2^ = 0.074; *B* = −0.191, *t* = −2.000, *p = .048) and betweenness (*F* = 2.415, *p = .038, *R* ^2^ = 0.066; *B* = 0.304, *t* = 3.141, *p = .002) of the right posterior superior temporal gyrus (pSTG) within the language network. In Figure 2c, the CTQ score was negatively related to efficiency of the right pSTG (*r* = −0.190, *p = .011) and positively associated with betweenness of the right pSTG (*r* = 0.201, *p = .007), respectively.

### 3.4 Mediation analysis

**Figure 3:**
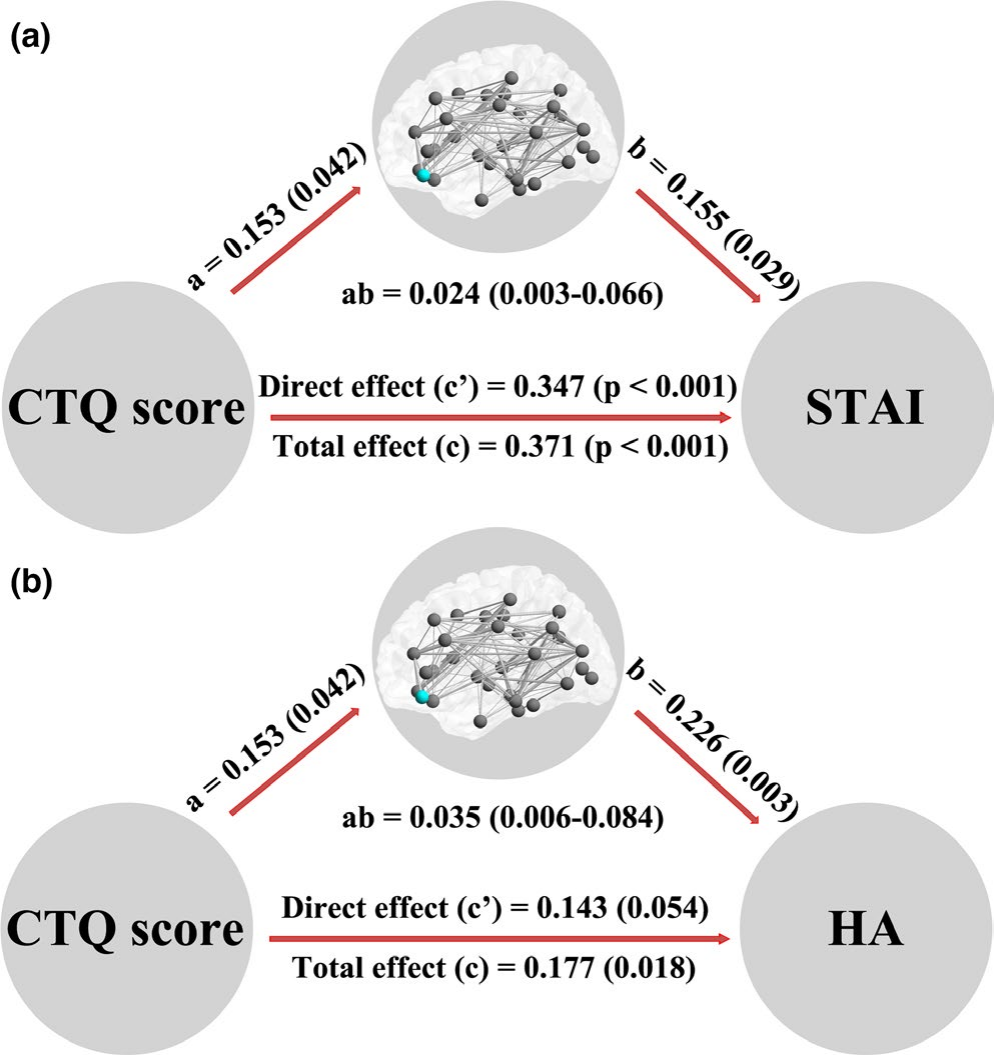
Significant mediation effects in the environment–brain–behavior pathway. We found that efficiency of the right medial OFC within MSN was a significant mediator between CTQ score and STAI/HA. Significant indirect effects were labeled with path coefficients and 95% confidence intervals, while the direct and total effects were labeled with path coefficients and *p* values. In figure a, there were significant positive effects from CTQ score to STAI score (*c* = 0.371, *c*′ = 0.347, *p* < .001), from CTQ score to efficiency of the right medial OFC (*a* = 0.153, *p* = .042), and from efficiency of the right medial OFC to STAI score (*b* = 0.155, *p* = .029); the ratio of indirect to total effect was 0.0636. In figure b, there were significant positive effects from CTQ score to harm‐avoiding score (*c* = 0.177, *p* = .018), from CTQ score to efficiency of the right medial OFC (*a* = 0.153, *p* = .042), and from efficiency of the right medial OFC to harm‐avoiding score (*b* = 0.226, *p* = .003); the ratio of indirect to total effect was 0.1945. CTQ: Childhood Trauma Questionnaire; HA: harm avoiding; MSN: morphometric similarity network; OFC: orbitofrontal cortex; STAI: State‐Trait Anxiety Inventory

Only efficiency of the right medial OFC within MSN was found as a significant mediator between CTQ score and STAI score/harm‐avoiding score (Figure 3). Significant indirect effects were labeled with path coefficients and 95% confidence intervals, while the direct and total effects were labeled with path coefficients and p values. In Figure 3a, there were significant positive effects from CTQ score to STAI score (*c* = 0.371, *c*′ = 0.347, *p* < .001), from CTQ score to efficiency of the right medial OFC (*a* = 0.153, *p* = .042), and from efficiency of the right medial OFC to STAI score (*b* = 0.155, *p* = .029); the ratio of indirect to total effect was 0.0636. In Figure 3b, there were significant positive effects from CTQ score to harm‐avoiding score (*c* = 0.177, *p* = .018), from CTQ score to efficiency of the right medial OFC (*a* = 0.153, *p* = .042), and from efficiency of the right medial OFC to harm‐avoiding score (*b* = 0.226, *p* = .003); the ratio of indirect to total effect was 0.1945.

## 4 DISCUSSION

In this study, we found main effects of childhood trauma experience on anatomic connectivity of the right medial OFC and functional connectivity of the right pSTG. Anatomic connectivity of the right medial OFC was an outstanding mediator supporting the relationship between childhood adversity and anxiety/harm‐avoiding personality. On the other hand, we showed genotype × CTQ score interactions on functional connectivity of the right frontoparietal network as well as anatomic connectivity of the right paracentral lobule and the right caudal ACC, suggesting genetically modulation on environmental influences.

### 4.1 Childhood adversity leads to experience‐dependent brain modification of anatomic connectivity in the medial OFC

We reported the main effect of the CTQ score on efficiency of the right medial OFC in MSN. MSN topology captures known cortical cytoarchitecture and is a novel approach to understanding how cortical connectivity underpins individual differences in psychological functions (Seidlitz et al., 2018). It provides morphometric similarity that represents developmental difference in cortical regions. Greater inter‐regional morphometric similarity is associated with stronger inter‐regional connectivity (Morgan et al., 2019). Increased efficiency of the right medial OFC in MSN was correlated with higher CTQ score, implying this region was more interconnected with other nodes in trauma‐exposed individuals. Additionally, the right medial OFC was an outstanding mediator that supported positive mediation effects of CTQ score on anxiety/harm‐avoiding personality. As a sensory cortex from cytoarchitectural classification, the medial OFC is critical for state representation such as threat processing and has specialized roles in mediating symptoms of anxiety disorders and depression (Schmaal et al., 2017; Sharpe et al., 2015). Damage in the medial OFC showed a long latent period to express defensive responses even in the absence of threat stimuli (Pujara et al., 2019). Exposure to childhood adversity is associated with alterations in the sensory systems that convey the aversive experience. Brain modifications of sensory systems may act as initiatory evidence of adaptable development in response to life stress. Enhanced anatomic connections of medial OFC may reveal oversensitiveness to negative stimuli and excessively defensive information processing in adults with childhood trauma experience, increasing the tendency of anxiety. Harm avoidance refers to behavioral inhibition for emotional process and is expressed as an innate tendency to pessimism. The OFC region, functionally responsible for response to emotional stimuli and reward anticipation, is associated with harm‐avoiding personality (Gardini et al., 2009). Enhanced anatomic connections of medial OFC may impact emotional processing and lead to the development of harm‐avoiding personality. Overall speaking, our findings suggest that childhood adversity leads to experience‐dependent brain modification of anatomic connectivity in the OFC, increasing risk for affective disorders.

### 4.2 Childhood adversity leads to experience‐dependent brain reorganization of functional connectivity within the language network

We found main effects of CTQ score on efficiency and betweenness of the right pSTG within the language network. Individual differences in children's understanding and expression skills mainly derive from the environment where they grow up. Excessive exposure to abuse and neglect has detrimental effects on a child's language cognition (Lum et al., 2018; Sylvestre et al., 2016). As a symmetrically distributed hub within the language network (Youssofzadeh & Babajani‐Feremi, 2019), STG is involved in language production and comprehension, and related to the dysregulation of functional connectivity in autism spectrum disorder (Lee et al., 2017). In our graph‐theoretical analyses of fMRI, connectivity efficiency of the STG was decreased while externally communication was enhanced in adults with high CTQ score. To some degree, these two contradictory changes disrupt functional connectivity within the language network. The experience‐dependent reorganization may impact emotional expression in trauma‐exposed individuals.

### 4.3 Interactive effects suggest contribution of the Met heterozygote to negative effects of childhood trauma on brain connectivity

Combining structural and functional analyses, our findings suggest modulation of the COMT Val158Met polymorphism on the relationship between early trauma experience and brain connectivity. In MSN analysis, we found significant genotype × CTQ score interactions on network properties of the right paracentral lobule and the right caudal ACC. The paracentral lobule is involved in planning, initiation, and execution of motor acts (Benito‐Leon et al., 2019). The ACC, cooperating with several other key brain regions, such as amygdala, OFC, insula, nucleus accumbens, and the frontal pole, affects emotion regulation during threat processing (Wymbs et al., 2020). In fMRI analysis, we found significant interaction on connectivity strength of the right frontoparietal network. The frontoparietal network includes main regions identified as supporting cognitive control and decision‐making process, involving the integration of information from the external environment with internal representations (Vincent et al., 2008). Negative information is associated with frontoparietal processing, enhancing our understanding of neural mechanisms of cognitive vulnerability to psychopathology (Westbrook et al., 2018). Overall, our findings show interactive effects on structural and functional connections involving motor performance, emotion regulation, executive function, and cognitive control.

Interestingly, significant correlations between childhood trauma and brain connectivity were only observed in the G/A genotype. The negative effects of childhood trauma on brain connections indicate the Met heterozygote to be risky for psychopathology in trauma‐exposed individuals. As we know, decreased enzymatic activity in the Met variation leads to increased synaptic dopamine concentrations. In a previous study, the Met heterozygote showed positive heterosis compared with other genotypes in the working memory tasks (Gosso et al., 2008). Potential mechanism is dopamine‐level‐dependent neurotrophic or neurotoxic effect. Consistent with the nonlinear relationship between the dopamine levels and neuronal activity (Bertolino et al., 2009; Qin et al., 2012; Seamans & Yang, 2004; Williams & Goldman‐Rakic, 1995), the effect of dopamine levels on structure and function of the brain has been commonly described as an inverted U‐shaped relationship (Bertolino et al., 2009; Goldman‐Rakic, 1998; Seamans & Yang, 2004; Williams & Goldman‐Rakic, 1995), in which the optimal level of extracellular dopamine can facilitate neuronal survival. In this study, we hypothesize that the Met heterozygote contributes to negative effects of childhood trauma on brain connectivity by showing a more suitable dopamine availability and a greater stress response to environmental adversity than other genotypes. Partly consistent with our findings, a number of studies in healthy samples suggest that carriers of the Met allele may be differentially affected by the environment stimuli, by showing larger sensitivity to negative mood states (Drabant et al., 2006; Smolka et al., 2005). The psychotic severity or affective reactivity to adversity is also greater in COMT Met allele carriers (Collip et al., 2011; Green et al., 2014; van Winkel et al., 2008). By contrast, no significant correlations between CTQ score and brain connectivity in the homozygote genotypes may demonstrate the protective effects of too low or too high extracellular dopamine on adversity‐related brain modifications by showing low sensitivity to environmental stimuli. However, the relatively low frequency of the Met homozygote in the sample may weaken our hypothesis. In future, it remains to be confirmed that whether the Met homozygote contributes to the protective effect of childhood trauma on brain connectivity in large‐sample neuroimaging genetic database.

Taken together, interactive effects between childhood trauma and the COMT Val158Met polymorphism suggest that the Met heterozygote contributes to negative effects of childhood trauma on brain connectivity involving motor performance, emotion regulation, executive function, and cognitive control. The Met heterozygote may act as genetically susceptible individuals, increasing the risk for developing psychopathology in adults with childhood trauma experience. Our findings help to shed light on the inverted U‐shaped modulation of dopamine on the relationship between childhood trauma and brain connectivity. Whereas cognitive vulnerability is a common trait in mental disorders, dopamine level may give rise to cognitive vulnerability depending on a range of endogenous factors and external stimuli (Bilder et al., 2004). Our findings may contribute to the pharmacogenetic studies in responses to cognitive interventions (e.g., trauma‐focused cognitive behavioral therapy, cognitive remediation therapy, and mindfulness training) that potentially improve brain connectivity to prevent or limit negative outcomes.

### 4.4 Limitations

This is an exploratory and preliminary study which has lots of limitations. Firstly, the effect size of multiple linear regression is relatively small. The majority of statistical results cannot pass the strict Bonferroni correction (**p < .001); then, an uncorrected threshold (*p < .05) was used to show the trend of significance. Although the assessment of statistical power needs to take into account research background and research type, as a neuroimaging genetic study, our results are still limited by the relatively small sample. In future, we will continue to increase the sample size to establish multicenter large‐sample neuroimaging genetic database. Next, we focus on the relationships among traumatic experience, the COMT Val158Met polymorphism, imaging phenotypes, and behavioral tendencies in nonclinical samples. The samples generally score below the cutoff for moderate‐to‐severe trauma. Our findings offer promising preliminary evidence that genetic and environmental factors are associated with brain connectivity, contributing to predicting the occurrence and development of psychopathology. Further researches need to elucidate the associations among traumatic experience, genotypes, impaired brain connectivity, and psychosis severity in clinical cohorts. Finally, given the current controversy about the validity and reliability of the candidate gene approach, we also need to investigate whether and how other dopaminergic system‐related genes modulate environment–brain–behavior pathway in future studies, even focusing on genome‐wide scans.

## 5 CONCLUSION

Our data suggest childhood trauma experience and the COMT Val158Met polymorphism are associated with abnormal brain connectivity, structurally and functionally, contributing to predicting the occurrence and development of psychopathology. On the one hand, childhood adversity leads to experience‐dependent modification of anatomic connectivity in the medial OFC, increasing tendency to anxiety and harm‐avoiding personality. Childhood adversity also gives rise to experience‐dependent reorganization of functional connectivity within the language network, impacting emotional expression in trauma‐exposed individuals. On the other hand, combining structural and functional analyses, interactive effects between childhood trauma and the COMT Val158Met polymorphism suggest that the Met heterozygote contributes to negative effects of childhood trauma on brain connectivity involving motor performance, emotion regulation, executive function, and cognitive control. Although our results are preliminary and limited, understanding the relationships among genetic factors, early life trauma, brain connectivity, and psychotic tendencies may have important clinical implications for the treatment or prevention. Early identification of individuals at risk, assessment of brain abnormality, and cognitive interventions may help to prevent or limit negative outcomes.

## CONFLICT OF INTEREST

The authors declared that the research was conducted in the absence of any commercial or financial relationships that could be construed as a potential conflict of interest.

## AUTHOR CONTRIBUTIONS

TT designed the study, analyzed the data, and wrote the manuscript. JL, JW, and GZ analyzed the data. DL, CW, JF, YZ, and DW collected the data. WZ provided feedback and edited the manuscript. All authors contributed to manuscript revision, read, and approved the submitted version.

## ETHICS APPROVAL AND SUBJECT CONSENT STATEMENT

The human experiment was approved by the Ethical Committee of Tongji Hospital of Tongji Medical College of Huazhong University of Science and Technology. All subjects gave written informed consent in accordance with the Declaration of Helsinki. All methods were carried out in accordance with approved institutional guidelines and regulations.

### Peer Review

The peer review history for this article is available at https://publons.com/publon/10.1002/brb3.1858.

## Supporting information

Fig S1

Fig S2

Table S1

Table S2

Table S3

Table S4

## Data Availability

The raw/processed data required to reproduce these findings cannot be shared at this time as the data also form part of an ongoing study.
