# Supplementary figures and images for: Effects of childhood trauma experience and COMT Val158Met polymorphism on brain connectivity in a multimodal MRI study
Source: Brain Behav. 2020 Sep 30;10(12):e01858. doi: 10.1002/brb3.1858 (PMC7749512; doi:10.1002/brb3.1858)

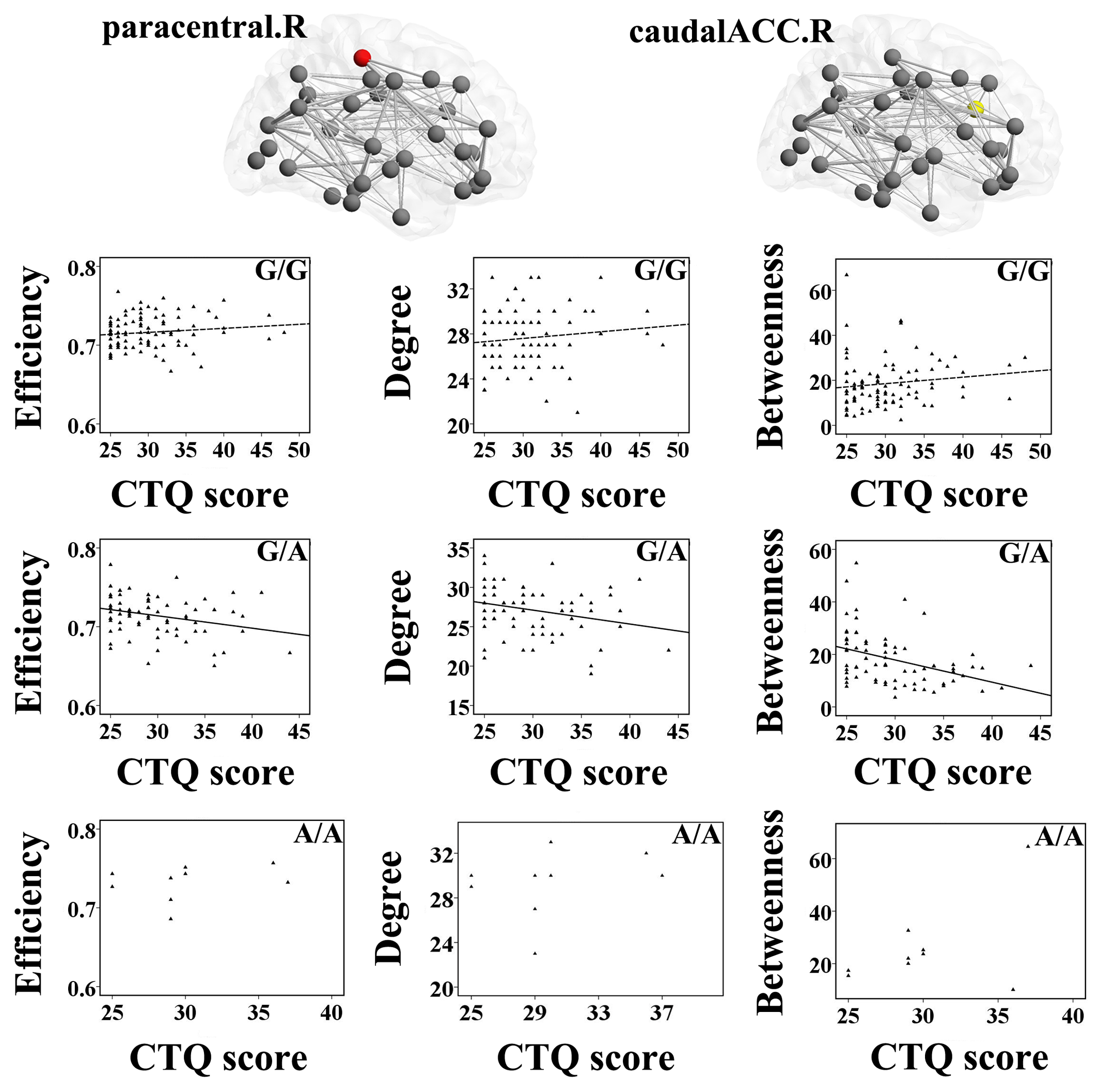

Supplement: Supplementary file 1 — Fig S1 [file BRB3-10-e01858-s001.tif]

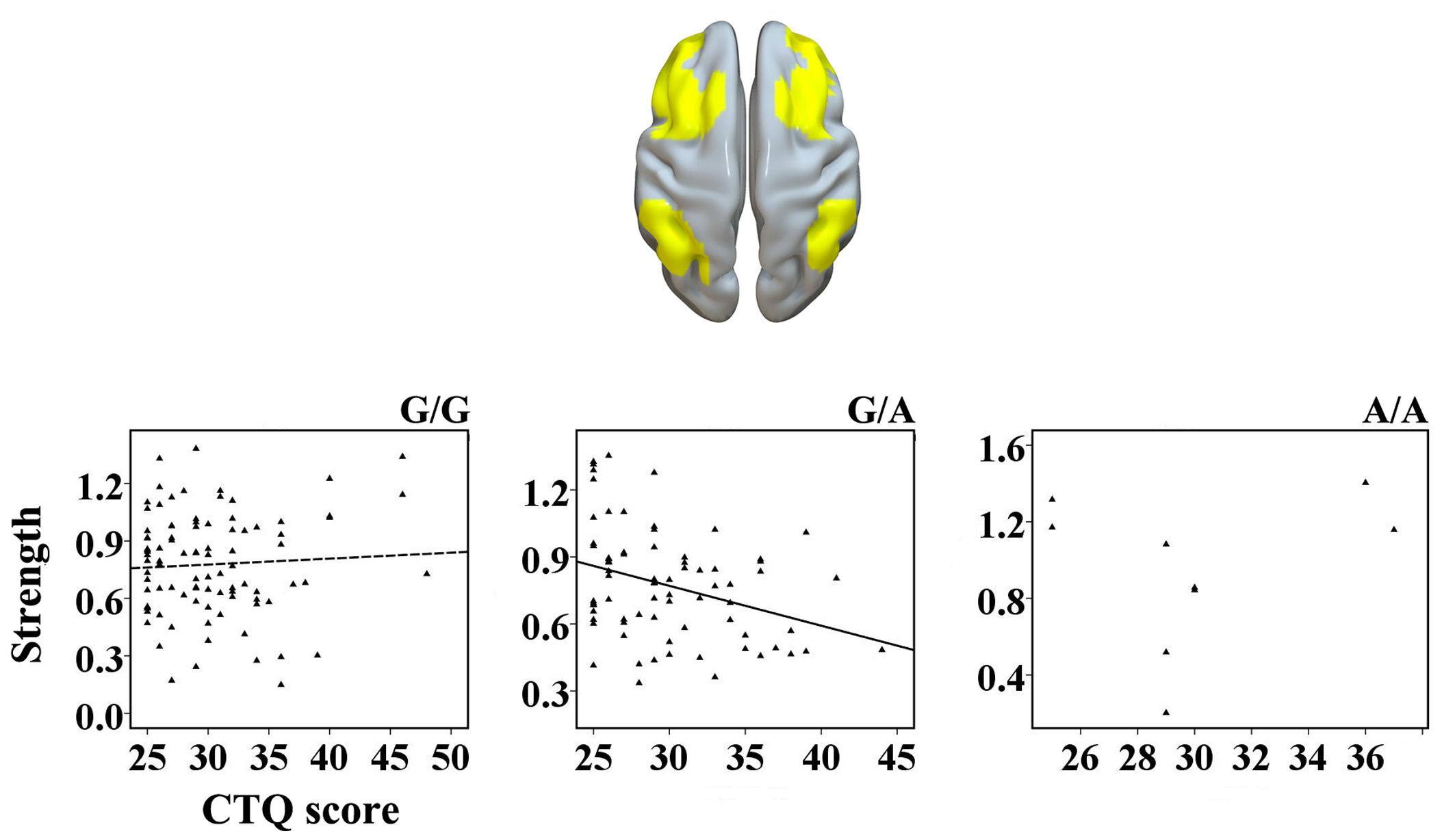

Supplement: Supplementary file 2 — Fig S2 [file BRB3-10-e01858-s002.tif]
