# Supplementary material for: Effects of childhood trauma experience and COMT Val158Met polymorphism on brain connectivity in a multimodal MRI study
Source: Brain Behav. 2020 Sep 30;10(12):e01858. doi: 10.1002/brb3.1858 (PMC7749512; doi:10.1002/brb3.1858)
Supplement: Supplementary file 3 — Table S1 [file BRB3-10-e01858-s003.docx]

Table S1 31 cortical regions per hemisphere in the Desikan–Killiany–Tourville labeling protocol.

| **Temporal lobe (medial)** |
| --- |
| Entorhinal cortex |
| Parahippocampal gyrus |
| Fusiform gyrus |
| **Temporal lobe (lateral)** |
| Superior temporal gyrus |
| Middle temporal gyrus |
| Inferior temporal gyrus |
| Transverse temporal gyrus |
| **Frontal lobe** |
| Superior frontal gyrus |
| Caudal middle frontal gyrus |
| Rostral middle frontal gyrus |
| Pars opercularis inferior frontal gyrus |
| Pars triangularis inferior frontal gyrus |
| Pars orbitalis inferior frontal gyrus |
| Lateral orbitofrontal gyrus |
| Medial orbitofrontal gyrus |
| Precentral gyrus |
| Paracentral lobule |
| **Parietal lobule** |
| Postcentral gyrus |
| Supramarginal gyrus |
| Superior parietal lobule |
| Inferior parietal lobule |
| Precuneus |
| **Occipital lobe** |
| Lingual gyrus |
| Pericalcarine cortex |
| Cuneus cortex |
| Lateral occipital cortex |
| **Cingulate cortex** |
| Rostral anterior |
| Caudal anterior |
| Posterior |
| Isthmus |
| **Insula** |
