# Supplementary material for: Effects of childhood trauma experience and COMT Val158Met polymorphism on brain connectivity in a multimodal MRI study
Source: Brain Behav. 2020 Sep 30;10(12):e01858. doi: 10.1002/brb3.1858 (PMC7749512; doi:10.1002/brb3.1858)
Supplement: Supplementary file 4 — Table S2 [file BRB3-10-e01858-s004.docx]

Table S2 30 regions of interest from Conn network cortical atlas.

| Regions | Montreal Neurological Institute coordinate (x, y, z) | | |
| --- | --- | --- | --- |
| **Default mode network** |  |  |  |
| Default mode.MPFC | 1 | 55 | -3 |
| Default mode.LP.L | -39 | -77 | 33 |
| Default mode.LP.R | 47 | -67 | 29 |
| Default mode.PCC | 1 | -61 | 38 |
| **Sensorimotor network** |  |  |  |
| Sensorimotor.Lateral.L | -55 | -12 | 29 |
| Sensorimotor.Lateral.R | 56 | -10 | 29 |
| Sensorimotor.Superior | 0 | -31 | 67 |
| **Visual network** |  |  |  |
| Visual.Medial | 2 | -79 | 12 |
| Visual.Occipital | 0 | -93 | -4 |
| Visual.Lateral.L | -37 | -79 | 10 |
| Visual.Lateral.R | 38 | -72 | 13 |
| **Salience network** |  |  |  |
| Salience.ACC | 0 | 22 | 35 |
| Salience. Anterior insula.L | -44 | 13 | 1 |
| Salience. Anterior insula.R | 47 | 14 | 0 |
| Salience.RPFC.L | -32 | 45 | 27 |
| Salience.RPFC.R | 32 | 46 | 27 |
| Salience.SMG.L | -60 | -39 | 31 |
| Salience.SMG.R | 62 | -35 | 32 |
| **Dorsal attention network** |  |  |  |
| Dorsal attention.FEF.L | -27 | -9 | 64 |
| Dorsal attention.FEF.R | 30 | -6 | 64 |
| Dorsal attention.IPS.L | -39 | -43 | 52 |
| Dorsal attention.IPS.R | 39 | -42 | 54 |
| **Frontoparietal network** |  |  |  |
| Frontoparietal.LPFC.L | -43 | 33 | 28 |
| Frontoparietal.PPC.L | -46 | -58 | 49 |
| Frontoparietal.LPFC.R | 41 | 38 | 30 |
| Frontoparietal.PPC.R | 52 | -52 | 45 |
| **Language network** |  |  |  |
| Language.IFG.L | -51 | 26 | 2 |
| Language.IFG.R | 54 | 28 | 1 |
| Language.pSTG.L | -57 | -47 | 15 |
| Language.pSTG.R | 59 | -42 | 13 |

ACC: anterior cingulate cortex; FEF: frontal eye fields; IFG: inferior frontal gyrus; IPS: intra parietal sulcus; L: left; LP: lateral parietal lobe; LPFC: lateral prefrontal cortex; MPFC: medial prefrontal cortex; PCC: posterior cingulate cortex; PPC: posterior parietal cortex; pSTG: posterior superior temporal gyrus; R: right; RPFC: rostral prefrontal cortex; SMG: supramarginal gyrus.
