# Supplementary material for: Effects of childhood trauma experience and COMT Val158Met polymorphism on brain connectivity in a multimodal MRI study
Source: Brain Behav. 2020 Sep 30;10(12):e01858. doi: 10.1002/brb3.1858 (PMC7749512; doi:10.1002/brb3.1858)
Supplement: Supplementary file 5 — Table S3 [file BRB3-10-e01858-s005.docx]

Table S3. Significant differences in mean nodal similarity between classes. The Bonferroni correction was used for multiple comparisons.

| (I) class | (J) class | mean difference (I-J) | significance |
| --- | --- | --- | --- |
|  |  |  |  |
| 1 | 2 | 0.006 | - |
|  | 3 | 0.136 | **p < 0.001 |
|  | 4 | -0.011 | - |
|  | 5 | 0.025 | - |
|  | 6 | -0.289 | **p < 0.001 |
|  | 7 | -0.160 | **p < 0.001 |
| 2 | 1 | -0.006 | - |
|  | 3 | 0.130 | **p < 0.001 |
|  | 4 | -0.017 | - |
|  | 5 | 0.018 | - |
|  | 6 | -0.295 | **p < 0.001 |
|  | 7 | -0.167 | **p < 0.001 |
| 3 | 1 | -0.136 | **p < 0.001 |
|  | 2 | -0.130 | **p < 0.001 |
|  | 4 | -0.147 | **p < 0.001 |
|  | 5 | -0.112 | **p < 0.001 |
|  | 6 | -0.425 | **p < 0.001 |
|  | 7 | -0.297 | **p < 0.001 |
| 4 | 1 | 0.011 | - |
|  | 2 | 0.017 | - |
|  | 3 | 0.147 | **p < 0.001 |
|  | 5 | 0.035 | - |
|  | 6 | -0.278 | **p < 0.001 |
|  | 7 | -0.150 | **p < 0.001 |
| 5 | 1 | -0.025 | - |
|  | 2 | -0.018 | - |
|  | 3 | 0.112 | **p < 0.001 |
|  | 4 | -0.035 | - |
|  | 6 | -0.314 | **p < 0.001 |
|  | 7 | -0.185 | **p < 0.001 |
| 6 | 1 | 0.289 | **p < 0.001 |
|  | 2 | 0.295 | **p < 0.001 |
|  | 3 | 0.425 | **p < 0.001 |
|  | 4 | 0.278 | **p < 0.001 |
|  | 5 | 0.314 | **p < 0.001 |
|  | 7 | 0.129 | **p < 0.001 |
| 7 | 1 | 0.160 | **p < 0.001 |
|  | 2 | 0.167 | **p < 0.001 |
|  | 3 | 0.297 | **p < 0.001 |
|  | 4 | 0.150 | **p < 0.001 |
|  | 5 | 0.185 | **p < 0.001 |
|  | 6 | -0.129 | **p < 0.001 |
