# Supplementary material for: Effects of childhood trauma experience and COMT Val158Met polymorphism on brain connectivity in a multimodal MRI study
Source: Brain Behav. 2020 Sep 30;10(12):e01858. doi: 10.1002/brb3.1858 (PMC7749512; doi:10.1002/brb3.1858)
Supplement: Supplementary file 6 — Table S4 [file BRB3-10-e01858-s006.docx]

Table S4. Significant differences in mean functional connectivity between networks. The Bonferroni correction was used for multiple comparisons.

| (I) network | (J) network | mean difference (I-J) | significance |
| --- | --- | --- | --- |
|  |  |  |  |
| Default mode | Sensorimotor | -0.194 | **p < 0.001 |
|  | Visual | -0.001 | - |
|  | Salience | 0.008 | - |
|  | Dorsal attention | -0.067 | *p = 0.013 |
|  | Frontoparietal | -0.5217 | **p < 0.001 |
|  | Language | -0.1987 | **p < 0.001 |
| Sensorimotor | Default mode | 0.194 | **p < 0.001 |
|  | Visual | 0.193 | **p < 0.001 |
|  | Salience | 0.202 | **p < 0.001 |
|  | Dorsal attention | 0.127 | **p < 0.001 |
|  | Frontoparietal | -0.327 | **p < 0.001 |
|  | Language | -0.004 | - |
| Visual | Default mode | 0.001 | - |
|  | Sensorimotor | -0.193 | **p < 0.001 |
|  | Salience | 0.009 | - |
|  | Dorsal attention | -0.065 | *p = 0.017 |
|  | Frontoparietal | -0.520 | **p < 0.001 |
|  | Language | -0.197 | **p < 0.001 |
| Salience | Default mode | -0.008 | - |
|  | Sensorimotor | -0.202 | **p < 0.001 |
|  | Visual | -0.009 | - |
|  | Dorsal attention | -0.074 | *p = 0.003 |
|  | Frontoparietal | -0.530 | **p < 0.001 |
|  | Language | -0.206 | **p < 0.001 |
| Dorsal attention | Default mode | 0.067 | *p = 0.013 |
|  | Sensorimotor | -0.127 | **p < 0.001 |
|  | Visual | 0.065 | *p = 0.017 |
|  | Salience | 0.074 | *p = 0.003 |
|  | Frontoparietal | -0.455 | **p < 0.001 |
|  | Language | -0.131 | **p < 0.001 |
| Frontoparietal | Default mode | 0.521 | **p < 0.001 |
|  | Sensorimotor | 0.327 | **p < 0.001 |
|  | Visual | 0.520 | **p < 0.001 |
|  | Salience | 0.529 | **p < 0.001 |
|  | Dorsal attention | 0.455 | **p < 0.001 |
|  | Language | 0.323 | **p < 0.001 |
| Language | Default mode | 0.198 | **p < 0.001 |
|  | Sensorimotor | 0.004 | - |
|  | Visual | 0.197 | **p < 0.001 |
|  | Salience | 0.206 | **p < 0.001 |
|  | Dorsal attention | 0.131 | **p < 0.001 |
|  | Frontoparietal | -0.323 | **p < 0.001 |
